# Current State of Evidence for Medication Treatment of Preschool Internalizing Disorders

**DOI:** 10.1155/2014/286085

**Published:** 2014-01-27

**Authors:** Justin A. Barterian, Erin Rappuhn, Erin L. Seif, Gabriel Watson, Hannah Ham, John S. Carlson

**Affiliations:** 401c Erickson Hall, Michigan State University, East Lansing, MI 48824, USA

## Abstract

Psychotropic medications are being prescribed off-label by psychiatrists to treat preschool children diagnosed with internalizing disorders. In this review, the current state of evidence is presented for medications used to treat preschool children (ages 2–5 year olds) diagnosed with anxiety and/or depressive disorders. Eleven studies were systematically identified for this review based on a priori criteria. Overall, the available literature revealed that studies addressing the medication treatment of internalizing disorders in preschoolers are extremely limited and represent relatively weak research methodologies. Given the increasing prevalence of the use of psychotropic medications to treat preschool children and the unique challenges associated with working with this population, it is imperative that mental health practitioners are aware of the current, albeit limited, research on this practice to help make informed treatment decisions. Suggestions about how to monitor potential costs and benefits in those unique cases in which psychopharmacological treatments might be considered for young children are given. Moreover, areas of additional research for this population are discussed.

## 1. Introduction

Gaps in the literature pertaining to the diagnosis and treatment of preschool internalizing mental health disorders have been frequently identified within the medical profession (e.g., [1–4]). One of those gaps is the off-label prescribing of psychotropic medications for preschool internalizing disorders. The scope of this controversial practice is still being closely monitored and further understanding and study are warranted given the potential effectiveness of this treatment approach within school-aged populations [5–8].

One study looking at the prescription practices within a Health Maintenance Organization reported a small proportion of preschool children (16%) with diagnosed behavioral or emotional problems as being prescribed a psychotropic medication [9]. Estimates indicate that less than 3% of all preschool children have been treated with a psychotropic medication, yet evidence suggests that this pattern has increased over time [10, 11]. This trend in prescribing appears to be especially true for antidepressants (i.e., tricyclic antidepressants and selective serotonin reuptake inhibitors), which were reported as the second most commonly prescribed medications for preschoolers behind psychostimulants more than a decade ago [12]. Ironically, the antidepressants most commonly prescribed to treat internalizing disorders in children (i.e., fluoxetine for depression/obsessive-compulsive disorder (OCD) for ages 8 and older, sertraline for OCD for ages 6 and older, and fluvoxamine for OCD for ages 8 and older) also hold the most serious type of prescription warning available (i.e., “black box” designation) by the Food and Drug Administration (FDA) [13] given the potential to increase suicidal thinking and behavior.

Anxiety and mood disorders (i.e., internalizing disorders) are the most common mental health conditions experienced by young children with prevalence rates at about 10% [14]. Preschool children with untreated internalizing disorders are likely to display symptoms throughout childhood. For example, research has demonstrated that children with depression in preschool are more likely to be depressed two years later [15]. The chronic nature of disorders that appear in early childhood is troublesome given that children diagnosed with internalizing disorders suffer significant challenges and problems associated with these disorders. For instance, children with OCD experience a low quality of life compared to their peers [16]. Moreover, evidence suggests that anxiety disorders can negatively impact an individual's level of educational attainment [17]. The costs associated with untreated internalizing disorders and conditions that are resistant to psychosocial interventions often leave prescribers and families in a quandary with respect to treatment options for young children experiencing chronic, persistent, and dysfunctional symptoms of anxiety and depression.

Even though few young children are prescribed psychotropic medicines (e.g., psychostimulants, selective serotonin reuptake inhibitors (SSRIs)), a paucity of research demonstrates that they may be beneficial for preschool children experiencing severe mental health conditions [6]. However, Scahill and colleagues [18] raised a number of issues pertaining to the costs and benefits of SSRIs within children and adolescents with major depression. Specifically, behavioral activation (e.g., impulsivity, disinhibition), self-harm, and suicidal ideation are all of significant concern. Safer and Zito's [19] findings indicated that children are two to three times more likely to exhibit side effects like disinhibition and gastrointestinal upset when compared to adults taking these medicines. Furthermore, in a retrospective chart review of 39 children under age 7 treated with SSRIs, eleven (28%) were reported to experience side effects (e.g., behavioral activation) severe enough to warrant discontinuation [20]. In sum, it is important to recognize that age plays a major role in the development and the seriousness of side effects that may be associated with SSRI treatment [4, 21].

Given the lack of knowledge pertaining to how these medicines may impact the rapidly maturing brains and bodies of young children, there is a great need to be overly cautious in using these medications within the treatment of preschool internalizing disorders [3, 22]. Best practice assessment and treatment guidelines advocate for a thorough employment of diagnostic procedures consistent with comprehensive methods used with older children [2, 6, 23]. Adherence to a number of important ethical considerations (e.g., explicit communication with families regarding the lack of approval by the Food and Drug Administration [13] for these medicines) is recommended when a failed psychosocial intervention leads to consideration of a medication trial for anxiety or depressive symptoms in young children [6].

The purpose of this paper is to review and summarize the current state of evidence regarding the efficacy and effectiveness of psychotropic medications with preschool children who have been diagnosed with an internalizing disorder. In addition, a close look at the reported adverse effects associated with these treatments and a critical look at the research methodologies reported in the literature are undertaken. Implications and suggestions for mental health practitioners based on the available research will be discussed. Three research questions were addressed in this critical review of the literature as follows.

1. What medications have been researched in the treatment of preschool internalizing disorders?
2. What is the state of evidence for the efficacy and effectiveness of these medications?
3. What side effects are reported for these medicines?

## 2. Methods

A comprehensive literature review was conducted in order to identify studies that examined the effectiveness of psychotropic medications for preschool children between the ages of 2 and 5 years old. Three databases were used including: Psyc Info, Psyc Articles, and Pub Med. In each database, the following terms were searched to identify relevant articles “preschool,” “toddler,” or “children,” in combination with “internalizing disorder,” “anxiety,” “depression,” “obsessive compulsive disorder,” “selective mutism,” “generalized anxiety disorder,” “separation anxiety disorder,” or “specific phobia,” in combination with “psychotropic medication,” “psychopharmacological treatment,” “anxiolytics,” “antidepressants,” “Selective Serotonin Reuptake Inhibitors,” “Fluoxetine,” “Fluvoxamine,” “Sertraline,” or “Citalopram.”

Studies that were written in English were selected. After identifying these studies, the authors went through to identify only those studies that described specific outcomes for preschool children. For example, if a study only reported mean improvement rates and only included a handful of preschoolers in a large sample that included older children, the study was discarded. This entire process was repeated independently to ensure that all relevant studies were found and included in the review.

### 2.1. Characteristics of Identified Studies

The search procedure identified 11 studies that specifically reported on the outcomes for preschool children who were prescribed a psychotropic medication to treat an internalizing disorder. All 11 studies identified focused on the use of psychotropic drugs for an anxiety disorder such as OCD, specific phobia, or selective mutism, a variant of social phobia [2]. The search procedure failed to identify any studies that reported results on the use of psychotropic medications for the treatment of unipolar depression specifically for preschool children. Studies that examined the treatment of bipolar disorder in preschool children, such as the examination of risperidone for the treatment of preschool onset bipolar disorder by Pavuluri and colleagues [24], were not included, as these were outside of the purview of this review. A majority of the studies (*n* = 7) found in this search focused on the use of psychotropic medications alone for the treatment of an anxiety disorder while four studies examined the use of a psychotropic drug in combination with other therapies. These psychosocial treatments included supportive psychotherapy, behavioral therapy, family therapy, psychodynamic music therapy, and school-based behavioral interventions. A total of three studies included a combination of psychotropic medications. Although 11 studies reported on the use of psychotropic medications for the treatment of internalizing disorders in preschool children, only a little more than half (*n* = 6) used standardized measures to monitor treatment and outcomes. With respect to research methodology, eight of the identified studies used a case study design, one used a multiple baseline single-case design, and two used a quasi-experimental small group design. Specific attention to the primary diagnosis, type of medication used, sample size and demographics, study design, and type of standardized outcome measures used within each study are highlighted in Table 1.

## 3. Results

Limited variability with respect to medication class of drugs used to treat preschool internalizing disorders was found in this review. SSRIs accounted for the majority of medications examined (*N* = 10 of 11 studies), with the predominant use of fluoxetine (*N* = 8 of the 11 studies) reported in the literature. The specific medications, targeted outcomes, methods employed, and reported findings are presented in the following paragraphs.

### 3.1. Selective Serotonin Reuptake Inhibitors

#### 3.1.1. Fluoxetine

Eight studies used fluoxetine to treat various disorders including selective mutism (*n* = 4), obsessive-compulsive disorder (*n* = 2), posttraumatic eating disorder/fear of feeding (*n* = 1), and specific phobia (*n* = 1). However, even though fluoxetine is the most researched psychotropic medication for preschool children with internalizing disorders, only two studies employed methodologies more rigorous than case study designs. A brief overview of each of all of these studies with specific attention on intended and adverse effects is presented by disorder.

*(1) Selective Mutism.* Dummit III and colleagues [25] conducted a quasi-experimental study examining the use of fluoxetine for children diagnosed with selective mutism. Despite enrolling children up to age 17, this study included two male and three female five-year-old children. Although this study was quasi-experimental in nature, it should be noted that a separate pretest/posttest analysis (i.e., *t*-test) was not completed specifically for preschool children. Therefore, only individual improvement data for the preschool children can be reported in this review. Prescriptions of fluoxetine for preschool children at the end of the study ranged from 10 mg/day to 20 mg/day. All five of these children displayed improvements on the Clinical Global Impression-Scale (CGI-S) completed by the prescribing physician. Despite the noted improvements, fluoxetine was discontinued with two of the five preschool children because of symptoms of behavioral disinhibition. The authors did not report any other child specific side effects for preschool children. Adverse events for the entire study sample included: difficulty falling asleep, jitteriness, headache, abdominal pain, decreased appetite, decreased arousing, drowsiness, irritability, and agitation.

Golwyn and Sevlie [26] reported on the use of fluoxetine with a four-year-old female with selective mutism. Dosage was started at two mg/day and gradually increased to 16 mg/day. After four months of treatment at 10 mg/day, the child began to be calmer, smile more often, and was able to speak with office staff. However, after 10 months of treatment, the child was not speaking in the school setting. At this point, fluoxetine was discontinued and the child was started on phenelzine resulting in improvements in speaking behavior as described in a later section called “other medication treatments.” The authors did not report any side effects.

Harvey and Milne [27] examined the use of fluoxetine in combination with psychotherapy to treat a 5-year-old female with selective mutism. Psychotherapeutic services were provided individually to the child and to the family prior to beginning the medication trial. After six months of these services, the authors reported the child displayed minimal improvements. Therefore, the child was prescribed fluoxetine 20 mg/day starting at 2 mg/day with a gradual increase of 4 mg/day. After three months of treatment with fluoxetine at 20 mg/day the child was no longer displaying symptoms of selective mutism and was speaking frequently. The authors reported that side effects were minimal, but did not describe these adverse effects.

Wright and colleagues [28] reported on a case regarding a four-year-old female with selective mutism. The child presented as severely shy and refused to speak in several settings. Prior intensive psychotherapeutic treatments failed to produce any significant improvements in behavior. At this point, she was prescribed fluoxetine at four mg/day, which was increased to eight mg/day over a period of 12 days. After five days of treatment, the child began to talk in contexts that were comfortable to her. Furthermore, after 20 days of eight mg/day the child began to speak in several settings including school. She showed improvements on the internalizing symptoms domain of the Child Behavior Checklist (CBCL) from the first assessment period (*T* = 68—at risk) to the second (*T* = 60—not at risk). The authors reported that the child did not experience any serious side effects; however, it was noted that some of her pretreatment oppositional behaviors worsened. Specifics regarding these oppositional behaviors were not provided. 

*(2) Obsessive Compulsive Disorder.* Coskun and Zoroglu [29] conducted a retrospective quasi-experimental study that examined the use of fluoxetine to treat three male and three female preschool children with OCD by reviewing their medical records. Prescriptions of fluoxetine ranged from 5 mg to 15 mg/day. The CGI-S was used to determine baseline and outcome data. A Wilcoxon nonparametric paired *t*-test revealed that there was a significant mean improvement of CGI-S scale scores. Moreover, five out of the six children displayed individual improvements on the CGI-S. One participant did not experience any benefits from the medication at a dose of five mg/day and the medication was discontinued because of behavioral disinhibition. Behavioral disinhibition was a common side effect occurring in five of the six participants. Of note, one participant engaged in self-harming behaviors, but the specifics of these behaviors were not discussed. Other side effects included decreased appetite, weight loss, sleep difficulty, headache, abdominal pain, nightmares, drowsiness, tooth grinding, and upper respiratory tract infection.

Ercan et al. [30] reported on four case studies of the use of fluoxetine for the treatment of four preschool children, ages 2–5, with obsessive-compulsive disorder. Participants were three females and one male. Prescriptions of fluoxetine ranged from 5 mg/day to 20 mg/day. All participants improved with the use of fluoxetine as indicated by scores on the CGI and the CY-BOCS. The only side effect noted was behavioral disinhibition, which occurred in three out of the four children when receiving higher doses of fluoxetine. 

*(3) Specific Phobia.* Avci and colleagues [31] reported on a case study where fluoxetine was prescribed to treat a two-year-old girl who had an extreme phobia of driving in cars. Symptoms included panic attacks, trembling, heart palpitations, and sweating. After failed treatments with systematic desensitization, hydroxyzine (ten mg/day), and alprazolam (one to two mg/day), the child was started on five mg/day of fluoxetine. After two weeks at a dosage of five mg/day, the child's phobia fears dissipated and she was able to drive in a car without difficulty. When she was tapered off the medication after three months of treatment, specific phobia symptoms did not resurface. Avci and colleagues [24] did not report any side effects in their case study.

*(4) Feeding Anxiety.* Celik and colleagues [32] reported a case study completed with two 24-month old twin girls who were displaying severe fear of feeding due to previous medical complications. After treatment attempts with haloperidol (.5 mg/day) and behavior therapy failed, the children were started on five mg/day of fluoxetine. Behavior therapy was continued throughout the fluoxetine trial. After two months of treatment at five mg/day, the children began to feed without difficulty. After eight months, the fluoxetine was tapered and stopped. No side effects were reported throughout treatment. At the initial follow up after fluoxetine treatment, the children did not display any signs of feeding anxiety. At a three-year follow-up appointment, one child had developed separation anxiety and began showing symptoms of feeding anxiety again.

#### 3.1.2. Sertraline

Two studies examined the use of sertraline to treat anxiety disorders (i.e., selective mutism and OCD) in preschool children. One of these studies used a systematic single-case design methodology while the other used a case study design. Both studies reported increased functioning as a possible result of the sertraline treatment.

*(1) Selective Mutism.* Carlson et al. [33] examined the use of sertraline to treat five children with selective mutism using a multiple baseline single case design methodology. Two children in this study were five years old, while the remaining participants were school-aged. Prescriptions of sertraline ranged from 50 mg/day to 100 mg/day. Both preschool children experienced an improvement in symptoms based on several outcome measures including Goal Attainment Scaling (GAS) and scores on the Child Behavior Checklist (CBCL). Additionally, both children showed increased frequency of speech during direct observations. The parents of both preschool children “strongly agreed” that the sertraline treatment was an acceptable intervention for their child's symptoms. Despite the positive results, the authors suggest that caution is needed when interpreting these results, because of the limitations of the methodology. Only minimal side effects were noted for all of the study participants; however, one preschool child did develop insomnia at 100 mg/day of sertraline.

*(2) Obsessive Compulsive Disorder.* O. Oner and P. Oner [34] examined the use of sertraline to treat three children with OCD between the ages of 4 and 5. The first child was prescribed 25 mg/day of sertraline, which was increased to 50 mg/day after two weeks of initial treatment. However, risperidone was added to the treatment regimen because of behavioral disinhibition, which was likely a side effect of the sertraline. After 9 months of treatment, the symptoms that the child experienced diminished as assessed using the Children's Yale Brown Obsessive Compulsive Scale (CY-BOCS). After tapering the medication over a period of three months, the OCD symptoms did not return. The second child was also prescribed 25 mg/day of sertraline. This child also developed behavioral disinhibition and mild anorexia and was prescribed 0.5 mg/day of risperidone to treat the behavioral symptoms. After six months of sertraline treatment, the child was symptom free as rated on the CY-BOCS. However, the symptoms reoccurred after the medication was withdrawn for a month. The child was put back on 25 mg/day sertraline and symptoms dissipated. The third child was prescribed 25 mg/day of sertraline. Her symptoms disappeared after eight weeks of treatment as measured by the CY-BOCS and no side effects were reported.

### 3.2. Other Medication Treatments

#### 3.2.1. Phenelzine (Monoamine Oxidase Inhibitors; MAOIs)

Golwyn and Sevlie [26] prescribed 7.5 mg of phenelzine three times per day after a failed ten-month trial of fluoxetine for the treatment of selective mutism in a five-year old female child. After receiving phenelzine for three weeks, the child began to show affection towards a babysitter and initiated conversation with a nurse at the psychiatrist's office. After six weeks of treatment, the phenelzine was increased and she was put on an alternating schedule of 30 mg/day and 22.5 mg/day. At this point, she began speaking to other classmates as well as her teacher. Moreover, she started at a new school and was able to speak in front of the class. After 28 weeks of treatment on phenelzine, the child's dosage was tapered over a time frame of six months. Mutism symptoms did not reappear. The authors reported that the only side effects observed were insomnia, which was treated with clonazepam 0.25 mg/day, and slight weight gain. Despite these overall positive results, this medication and others MAOIs require considerable dietary restrictions and modifications (e.g., foods high in tyramine such as cheese) that may be particularly challenging for young children to follow [35].

#### 3.2.2. Buspirone (Azapirone)

Hanna et al. [36] reported on a case study in which they used buspirone, an azapirone, in order to treat a 4-year-old male diagnosed with anxiety symptoms related to feeding, which were possibly the result of pharyngeal dysphagia. The authors prescribed buspirone instead of an SSRI, because it was determined to be less likely to cause adverse gastrointestinal side effects. Buspirone was administered at 2.5 mg twice per day. After 1 week, the child began to use utensils and began eating more frequently and without prompting. After 8 weeks of treatment, the dosage was raised to 5 mg twice per day. The child continued to eat and gain weight after the dosage change. At this point in treatment, a third, mid-day, dose of 2.5 was added to promote eating while at school, and his food intake at school increased. When the child's parents removed him from the medication for two weeks without tapering, he began to show similar symptoms of feeding anxiety. Buspirone was reintroduced and the child's symptoms dissipated. The authors reported that the only side effect the child experienced was mild insomnia.

## 4. Discussion

### 4.1. Efficacy of Psychotropic Medications

The purpose of this article was to systematically review the current state of the literature pertaining to the use of psychotropic medications with preschool children experiencing internalizing disorders. Overall, the review found a paucity of information regarding the efficacy of psychopharmacological treatment for internalizing disorders in preschool children. Moreover, much of the data that exists is reported in unsystematic case studies, calling into question the generalizability of the data. Distressingly, none of the identified studies addressed psychopharmacological treatments for preschool children with depression. This is concerning given the debilitating and chronic nature of depression in preschool children [15, 37].

Despite the lack of rigorous research methodologies, SSRIs are the most studied medication family for preschool children with internalizing disorders. This makes sense given SSRIs are typically the first line of psychopharmacological treatment for older children and adults with internalizing disorders. In the research, fluoxetine was the most commonly prescribed followed by sertraline. Since only two studies examined the use of sertraline, it is clear, to date, that there is very little research to support its use with this population. Research data on other classes of medications, such as MAOIs and anxiolytics (i.e., azapirones), is extremely scarce.

### 4.2. Adverse Effects

SSRIs hold special warnings of suicidality for adolescents and young adults, and the downward extension of these possible side effects to preschool children has not been systematically investigated [6]. Only one study in this review [29] noted that self-harming behavior occurred; however, the unsystematic designs often used to evaluate medication outcomes in the available literature call into question whether this concern was monitored in all of the reviewed studies. A concerted effort by mental health practitioners and researchers alike should be made to systematically evaluate whether young children frequently experience this serious side effect.

Several mild to moderate short-term side effects were noted. The side effects experienced by preschool children appear to be similar to the adverse events experienced by older children. These side effects included upset stomach, headache, teeth grinding, insomnia, and behavioral disinhibition. Behavioral disinhibition occurred frequently throughout the identified studies, and this concern should be closely monitored in practice and research alike. None of the studies reviewed examined the longitudinal effects of preschool children taking a psychopharmacological medication. This lack of information continues to highlight significant ethical concerns about this practice.

### 4.3. Implications for Mental Health Practitioners

In sum, the use of psychotropic medications in the treatment of preschool internalizing disorders requires considerable care, caution, and concern. A thorough diagnostic assessment including a history of failed psychosocial treatment is essential. Gleason and colleagues [6] provide algorithms to help clinicians make determinations when treating preschool children with mental health problems. If potential benefits of a medication trial are evidently documented to outweigh the possible effects of continued symptoms and dysfunction, then it is imperative that the prescribing physician clearly identifies the target behaviors for treatment, as well as possible side effects. Such target symptoms in young children experiencing internalizing disorders might include: frequency of speech in cases of selective mutism, frequency/time of compulsions in children with OCD, and ratings of mood in children with depression. Practitioners may find http://www.schoolpsychiatry.org/ and http://www.psychiatry.org/ helpful for measures of effectiveness and side effects that may be applicable for preschool children. Mental health professionals should choose assessment tools that are efficient and reliable like Clinical Global Improvement (CGI) ratings which involve a collection of global perceptions of improvement across time, usually from the prescriber, the parent, and/or caregiver. Moreover, standardized and norm-referenced measures of behavior that have adequate reliability and validity data and are designed for preschool children such as the Selective Mutism Questionnaire (SMQ) [38] may be helpful for progress monitoring symptoms.

## 5. Conclusions

Overall, the results of this study indicate that the use of psychotropic medications with preschool children diagnosed with internalizing disorders is clearly in its infancy, and a significant amount of research needs to be undertaken to ensure that preschool children are receiving safe and effective care. Although all of the studies identified in the review reported positive results for most participants (e.g., reduced symptoms of anxiety), a majority of the studies published throughout the literature were case studies that lacked methodological rigor. Additional research is needed to ensure that the positive findings identified in the reviewed articles are generalizable and apply across settings (e.g., school, home, and community).

### 5.1. Limitations

This study has several limitations, the most obvious of which include the minimal data available on the practice of prescribing psychotropic medications for preschool internalizing disorders. In addition, this review only discussed the literature that was published in peer-reviewed journals. It is possible that unpublished literature, such as doctoral dissertations was missed. Finally, only studies that were disseminated in English were reviewed, making it possible that other available data presented in different languages were excluded.

## Figures and Tables

**Table 1:** Characteristics of identified studies.

Author(s)	Publication year	Diagnoses addressed	Medication(s)	*N* and participant characteristics	Design	Concurrent treatment	Treatment measures
Avci et al. [31]	1998	Specific Phobia	Fluoxetine (SSRI)	*N* = 1 Female Age: 2.5	Case study	None	None
Carlson et al. [33]	1999	Selective Mutism	Sertraline (SSRI)	*N* = 2 Females and 1 Male Ages: 5	Single case design	None	GAS, CBCL, CGI, PQ, TRS, TEQ-P
Coskun and Zoroglu [29]	2009	Obsessive Compulsive Disorder	Fluoxetine (SSRI)	*N* = 3 Females and 3 Males Ages: 3–5	Quasi-experimental design	Risperidone (*n* = 1) Hydroxyzine (*n* = 1)	CGI-S
Celik et al. [32]	2007	Posttraumatic Eating Disorder/fear of feeding	Fluoxetine (SSRI)	*N* = 2 Females Ages: 2	Case study	Behavior therapy	None
Dummit III et al. [25]	1996	Selective Mutism	Fluoxetine (SSRI)	*N* = 3 Females and 2 Males Age: 5	Quasi-experimental design	Supportive psychotherapy	CGI
Ercan et al. [30]	2012	Obsessive Compulsive Disorder	Fluoxetine (SSRI)	*N* = 3 Females and 1 Male Ages: 2–5	Case study	None	CGI CY-BOCS
Golwyn and Sevlie [26]	1999	Selective Mutism	Phenelzine (MAO-I) Fluoxetine (SSRI)	*N* = 1 Female Age: 4	Case study	Clonazepam in combination with Phenelzine (Insomnia)	None
Harvey and Milne [27]	1998	Selective Mutism	Fluoxetine (SSRI)	*N* = 1 Female Age: 5	Case study	Behavioral, family, and psychodynamic therapy; individual therapy for child; individual therapy for family members; occupational therapy; psychoeducation	None
Hanna et al. [36]	1997	Feeding Disorder of Infancy and Early Childhood/Anxiety	Buspirone (Azapirone)	*N* = 1 Male Age: 4	Case study	None	None
O. Oner and P. Oner [34]	2008	Obsessive Compulsive Disorder	Sertraline (SSRI)	*N* = 3 Females Ages: 4-5	Case study	Risperidone (*n* = 2)	CY-BOCS
Wright et al. [28]	1995	Selective Mutism	Fluoxetine (SSRI)	*N* = 1 Female Age: 4	Case study	School based behavioral intervention, behavior therapy, family therapy	CBCL, PSI, VABS
